# Supplementary material for: Simulation of Vancomycin Exposure Using Trough and Peak Levels Achieves the Target Area under the Steady-State Concentration–Time Curve in ICU Patients
Source: Antibiotics (Basel). 2023 Jun 27;12(7):1113. doi: 10.3390/antibiotics12071113 (PMC10376485; doi:10.3390/antibiotics12071113)
Supplement: Supplementary file 1 [file antibiotics-12-01113-s001.zip › antibiotics-2441498-supplementary.pdf]

Table S1. Optimal cut-off values related to achievement rate of the target AUC at the follow-up TDM.

	AUC	Cut-off	Sensitivity	Specificity	PPV	NPV
Change in CCr between initial and follow-up TDM	0.63	18.1	0.77	0.50	0.76	0.58
Days from initaial TDM to follow-up TDM	0.66	4	0.77	0.48	0.70	0.57

Abbreviations: AUC, area under the curve; CCr, creatinine clearance; PPV, positive predictive value; NPV, negative predictive value; TDM, therapeutic drug monitoring.

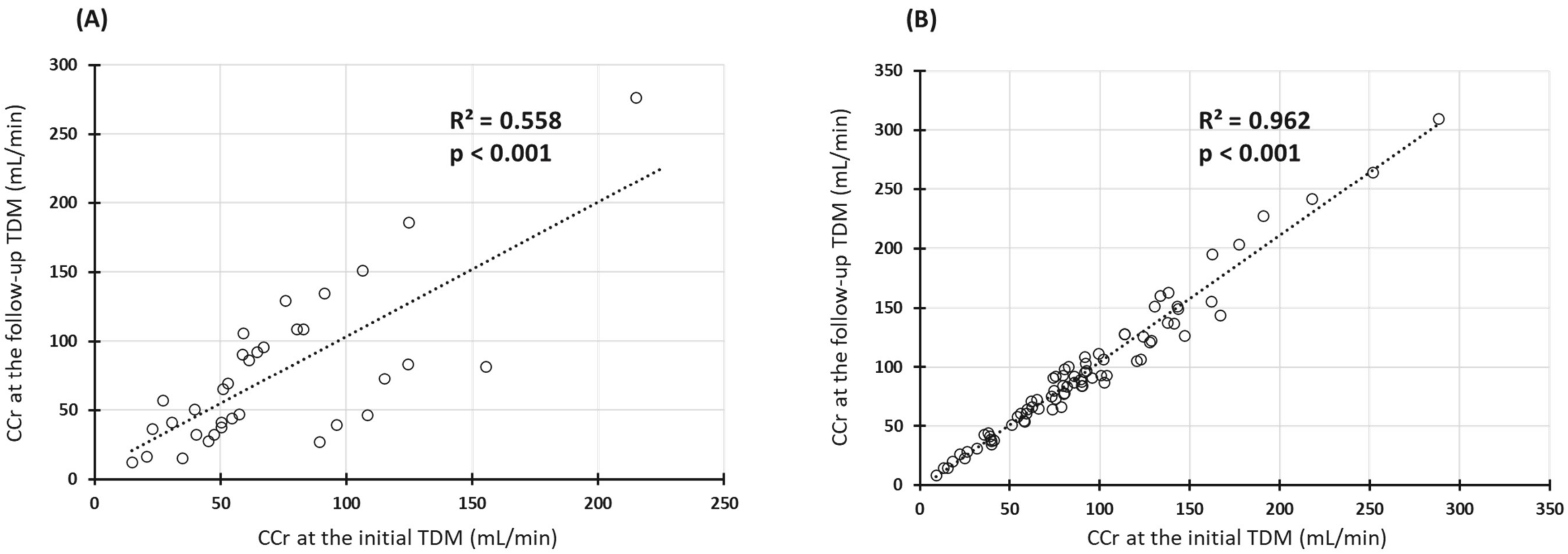

Figure S1. ***Comparison of CCr at the initial TDM and follow-up TDM.*** (**A**) With-change group (20% or more increase or decrease in CCr). (**B**) Without-change group (20% less than increase or decrease in CCr). $p < 0.05$ was considered statistically significant. Abbreviations: CCr, creatinine clearance; TDM, therapeutic drug monitoring.
